# Cherubism: best clinical practice

**DOI:** 10.1186/1750-1172-7-S1-S6

**Published:** 2012-05-24

**Authors:** Maria E Papadaki, Steven A Lietman, Michael A Levine, Bjorn R Olsen, Leonard B Kaban, Ernst J Reichenberger

**Affiliations:** 1 Department of Oral and Maxillofacial Surgery, Massachusetts General Hospital, Harvard School of Dental Medicine, Boston, MA, USA; 2 The Departments of Orthopaedic Surgery and Biomedical Engineering, Cleveland Clinic Lerner Research Institute, Cleveland, OH, USA; 3 Division of Endocrinology and Diabetes, The Children’s Hospital of Philadelphia and Department of Pediatrics, University of Pennsylvania School of Medicine, Philadelphia, PA, USA; 4 Department of Developmental Biology, Harvard School of Dental Medicine, Boston, MA, USA; 5 Department of Oral and Maxillofacial Surgery, Massachusetts General Hospital, Harvard School of Dental Medicine, Boston, MA, USA; 6 University of Connecticut Health Center, Department of Reconstructive Sciences, Center for Regenerative Medicine and Skeletal Development, Farmington, CT, USA

## Abstract

Cherubism is a skeletal dysplasia characterized by bilateral and symmetric fibro-osseous lesions limited to the mandible and maxilla. In most patients, cherubism is due to dominant mutations in the *SH3BP2* gene on chromosome 4p16.3. Affected children appear normal at birth. Swelling of the jaws usually appears between 2 and 7 years of age, after which, lesions proliferate and increase in size until puberty. The lesions subsequently begin to regress, fill with bone and remodel until age 30, when they are frequently not detectable.

Fibro-osseous lesions, including those in cherubism have been classified as quiescent, non-aggressive and aggressive on the basis of clinical behavior and radiographic findings. Quiescent cherubic lesions are usually seen in older patients and do not demonstrate progressive growth. Non-aggressive lesions are most frequently present in teenagers. Lesions in the aggressive form of cherubism occur in young children and are large, rapidly growing and may cause tooth displacement, root resorption, thinning and perforation of cortical bone.

Because cherubism is usually self-limiting, operative treatment may not be necessary. Longitudinal observation and follow-up is the initial management in most cases. Surgical intervention with curettage, contouring or resection may be indicated for functional or aesthetic reasons. Surgical procedures are usually performed when the disease becomes quiescent. Aggressive lesions that cause severe functional problems such as airway obstruction justify early surgical intervention.

## Disease name and definition

Cherubism (MIM ID# 118400) or multilocular cystic disease of the jaws was first recognized as a separate entity in 1933 by William A. Jones in a family with several affected members [1,2]. He designated the descriptive name “cherubism” because “the full round cheeks and the upward cast of the eyes give the children a peculiarly” cherubic appearance [2,3]. Because this name so accurately captured the clinical features of the disease, it became the standard nomenclature.

Cherubism is defined by the appearance of symmetrical, multilocular, expansile radiolucent lesions of the mandible and/or the maxilla that typically first appear at the age of 2 to 7 years. Swelling of submandibular lymph nodes in the early stages contributes to the fullness of the face. As the soft fibrous dysplastic tissue in the lesions expands the protuberant masses can infiltrate the orbital floor and cause the characteristic upward tilting of the eyes, exposing the sclera below the iris. Cherubism lesions are limited to the jaws and in most cases the dysplastic expansile masses begin to regress with the onset of puberty.

Grading systems for cherubism have been suggested to describe location and severity of lesions. The first system distinguished Grade 1: fibro-osseous bilateral and symmetrical expansions in the rami of the mandible; Grade 2: more severe involvement of the ramus and body of the mandible and the tuberosity region of the maxillae; and Grade 3: involvement of maxilla and mandible in their entirety with considerable facial deformity [4,5]. Raposo-Amaral simplified the grading system of Motamedi [6] and added a 6^th^ Grade to describe the involvement of the orbits [7] (Table 1). While some authors use this classification system to describe the extent of lesions, other authors do not use any grading system because the expression of cherubism in each patient is unique. Important for the clinician is the biologic/clinical behavior of the lesions in each patient: rate of growth, size, cortical bone perforation or thinning, tooth displacement and the functional deficits.

**Table 1:** Cherubism grading system according to Motamedi (1998) and Raposo-Amaral (2007)

Grade I Lesions of the mandible without signs of root resorption	Class 1	solitary lesion of the mandibular body
	Class 2	multiple lesions of the mandibular body
	Class 3	solitary lesion of the ramus
	Class 4	multiple lesions of the rami
	Class 5	lesions involving the mandibular body and rami
Grade II Lesions involving the mandible and maxilla without signs of root resorption	Class 1	lesions involving the mandible and maxillary tuberosities
	Class 2	lesions Involving the mandible and anterior maxilla
	Class 3	lesions involving the mandible and entire maxilla

Grade III Aggressive lesions of the mandible with signs of root resorption	Class 1	solitary lesion of the mandibular body
	Class 2	multiple lesions of the mandibular body
	Class 3	solitary lesion of the ramus
	Class 4	multiple lesions of the mandibular rami
	Class 5	lesions involving the mandibular body and rami

Grade IV Lesions involving the mandible and maxilla and showing signs of root resorption	Class 1	lesions involving the mandible and maxillary tuberosity
	Class 2	Lesions involving the mandible and anterior maxilla
	Class 3	lesions involving the mandible and entire maxilla

Grade V The rare, massively growing, aggressive, and extensively deforming juvenile cases involving the maxilla and mandible, and may include the coronoid and condyles		

Grade VI The rare, massively growing, aggressive, and extensively deforming juvenile lesions involving the maxilla, mandible and orbits		

## Epidemiology

Cherubism is a very rare disorder with only an estimated 300 cases reported in the literature. Because of its rarity, it is difficult to determine a disease frequency for this disorder. Cherubism affects males and females with equal frequency and has been reported in patients of all racial and ethnic backgrounds. Unequal penetrance between males and females should be considered a historical artifact, which is based on misinterpretation of a thorough clinical investigation [8,9]. The delayed disease onset and misdiagnosis of adult patients with a mild form of cherubism may have contributed to this misconception.

## Clinical description

The hallmark of cherubism is the development of symmetrical multilocular radiolucent expansile lesions in the mandible and/or the maxilla, which typically first appear at the age of 2 to 7 years. Submandibular and cervical lymph nodes are enlarged during the early stages of cherubism. Severity of the disease phenotype is highly variable, even within a family. Patients with a mild form of cherubism may develop only small symmetric lesions in the mandible. The first radiographic signs of cherubism are usually found in the region of the mandibular angle. These radiolucent lesions are asymptomatic but may affect development or eruption of permanent molars. The more progressive form of cherubism manifests with multiple symmetrical lesions in the mandible or involves the mandible and maxilla with singular or multiple lesions (see also Table 1).

Although, cherubism lesions are usually limited to the mandible and the maxilla, there are rare reports of involvement of the zygomatic arches and condyles [6,10]. Lesions in patients with the progressive form of cherubism result in extensive bone resorption and leave only a fenestrated shell of cortical bone. Fibrous tissue masses can expand the cortical bone and lead to facial swelling. When expansile fibrous tissue masses invade the floor and walls of the orbits they can cause upward tilting or displacement of the globes.

Most cases of cherubism regress spontaneously after puberty. There are rare instances when lesions in suspected cherubism patients are persist or actively grow in young adults [11,12].

## Extracranial involvement

As noted above, cherubism is typically limited to the craniofacial region. However, there are three reports in the literature that refer to involvement of the ribs. A 17-year-old girl from a family with 3 affected cherubism patients showed symptomless non-expansile lesions at the anterior ends of her ribs [13]. An 8-year-old boy presented with typical bilateral facial swelling and radiographic evidence of cherubism. The expansile growth invaded zygomatic bones bilaterally and multiple radiolucent lesions in the anterior ends of all ribs were found [10]. Similar lesions in ribs were radiographically detected in a 6-year-old patient with cherubism [14]. The reported cystic lesions of the ribs were asymptomatic in these cases and no follow-up has been reported.

Cherubism with co-expressed craniosynostosis and clubbed fingers has been described in a single family [15,16], However, it is not clear whether the appearance of these phenotypes is coincidental or associated with cherubism. Extracranial involvement is extremely rare and most cases have not been confirmed by genetic testing.

## Ocular involvement

The characteristic upward gaze of patients with cherubism provides the basis for the naming of the disease [1,2]. In more severe forms of cherubism, the fibro-osseous tissue extends into the inferior and/or lateral orbital walls. Physical displacement of the globe and retraction of the eyelids result in exposure of a rim of the sclera beneath the iris. The disease may also invade the retrobulbar spaces of the orbits and cause displacement of the optic nerves and proptosis [17]. The orbital effects of cherubism are due to this displacement and not to direct invasion of the globe and surrounding extraocular muscles. In one report, bilateral orbital floor tumor masses developed after general post-pubertal regression of the disorder [18]. At age 27 years the patient complained of reduced mobility of the eyes. Displacement of the globes was caused by multilocular bony tumors filled with a jelly-like tissue. In another case of a 27-year-old woman, the orbital lesions caused optic nerve dysfunction with decreased contrast sensitivity [19]. Ahmadi and colleagues describe a more severe case of orbital involvement where the patient, at 31 years of age, lost vision due to optic neuropathy, macular striae and scarring caused by compression of the globe [20]. These reports indicate that continued ophthalmologic supervision is mandatory even long after post-pubertal regression of cherubism lesions in the maxillae.

## Respiratory involvement

Respiratory problems are frequently absent but occasionally manifest as upper airway obstruction caused by backward displacement of the tongue [21] or obliteration of the nasal airway. These findings may lead to mouth breathing, snoring, chronic nasal infection and obstructive sleep apnea [22]. Nasopharyngoscopy, if possible, and an overnight polysomnogram should be obtained if concerns regarding a sleep disorder arise. Treatment could include continuous positive airway pressure [21], although this may not be possible because of the anatomy. Surgical intervention to alleviate nasal airway obstruction and tongue displacement or tracheotomy [22] may be necessary. There is one extreme case of cherubism in the literature that describes an 8-year-old boy with airway obstruction who died from consequent pulmonary infections, aspiration and septicemia [23].

## Dental impact

The impact of cherubism lesions on development and eruption of the primary and permanent dentition varies depending on the time of onset and severity of the expansile lesions. The arrangement of primary teeth can be disturbed [24,25]. Disruption of the secondary dentition can include absent teeth (mostly molars), rudimentary development of molars, abnormally shaped teeth, partially resorbed roots or delayed and ectopically erupting teeth [24,26]. Tooth extraction may be needed, especially if teeth are “free-floating” in cherubism lesions [6] or if they become ectopically impacted [27]. In more severe instances, children may require prostheses that need to be adjusted as the child grows or the swelling within the oral cavity changes. A dental prosthesis may improve the ability to chew and increase the self-esteem of the child. Orthodontic treatment is appropriate after growth is completed and when cherubism is regressing.

## Inflammatory aspects

In the early stages of disease, patients may present with lymph node enlargement. While most case reports of children with cherubism describe lymphadenopathy, this finding has not been monitored systematically and the natural history is not known. Early reports suggest that submandibular lymph nodes are enlarged during swelling of the lower portions of the face while upper cervical lymph nodes are involved when maxillary swelling occurs [2,10]. In the past, cherubism has not been considered an inflammatory bone disorder, but recent evidence in mouse models points to the possibility that it is indeed an autoinflammatory disease [28,29]. SH3BP2 is required for functional B-cell receptor (BCR) signaling [30]. In mouse models with mutant or ablated *Sh3bp2* genes [28,30,31] there is a delayed B-cell response [31]. The ubiquitously expressed SH3BP2 protein has different functions in different immune cells [30,32-35]. However, in *Sh3bp2*^KI/KI^ mice, the cherubism mouse model, Ueki et al. found that inflammatory lesions develop independently of B- or T-cell involvement [28]. There are at least two mechanisms that account for the cherubism-like phenotype in these mice: 1) Inflammatory reactions caused by macrophages that produce high amounts of TNF-α and 2) bone resorption caused by hyperactive osteoclasts via activation of NFATc1 [28,36].

## Biochemical markers

Mineral metabolism is normal in patients with cherubism, and serum levels of calcium, parathyroid hormone (PTH), parathyroid hormone related peptide (PTHrP), calcitonin and alkaline phosphatase (ALP) are typically within normal range [37]. Urine markers of bone remodeling such as pyridinium and deoxypyridinium cross-linking, hydroxyproline and calcium/creatinine have been reported to be at the upper limits of normal in some children [37]. Serum levels for alkaline phosphate may be increased during the active stages of cherubism [24,38-40]. Serum phosphate may also be increased [41]. Biochemical analysis can differentiate cherubism from hyperparathyroidism, particularly in patients with brown tumors (epulis) of the jaw or patients with the hyperparathyroidism-jaw tumor syndrome (HPT-JT) with mutations in the *HRPT2* gene encoding parafibromin [42-44]. Data about TNF-α levels in serum of cherubism patients have not been published, but there is preliminary evidence in a small group of patients and age-matched controls that TNF-α is elevated in cherubism patients (EJR personal communication).

## Histology

**Figure 1:**
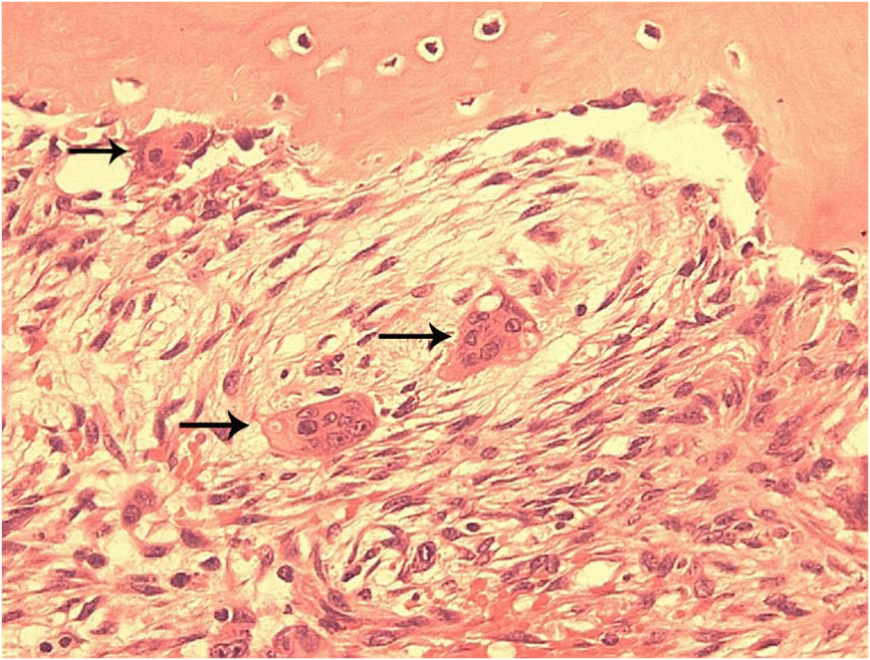
Typical histopathology of Cherubism. A histological section from a cherubism lesion demonstrates the typical finding of multinucleated osteoclast-like giant cells (arrows) near bone and within soft fibrous stroma.

Cherubism lesions resemble giant cell tumors because they contain many giant-cells and mononuclear or stromal cells (Figure 1). The fibrotic lesions are non-neoplastic. Cherubism cannot be diagnosed by histology alone because they are not distinguishable from other giant cell lesions of bone [45]. Details of histological findings at the various stages of cherubism are rarely described.

Chomette and colleagues describe 3 histologically, immunohistochemically and ultrastructurally distinct stages in cherubism lesions [46]. In the first, osteolytic stage the authors found numerous round, fusiform and multinucleated giant-cells. The giant osteoclast-like cells are tartrate resistant acid phosphatase (TRAP) positive. The tissue of the lesions is well vascularized. Fibroblastic cells with fewer giant-cells can be found in the periphery of the lesions. Hemosiderin, a breakdown product of hemoglobin and a sign of hemorrhage, is observed in endothelial cells and some surrounding fibroblasts.

The second stage is characterized by proliferative spindle cells, which the authors associated with a reparative stage. Fibroblastic nodules with central vessels dominate the lesion while some osteogenesis can be observed near the cortex of the bone. Newly formed bone matrix and osteoid should be seen.

The third stage is attributed to bone formation with cells staining positive for alkaline phosphatase (presumably differentiating osteoblasts) and high levels of ATPase (presumably associated with mineralizing matrix). The tissue contains more collagen fibers and fewer cells.

Ultrastructurally, the authors describe the multinucleated giant cells as osteoclasts, the ovoid cells as metabolically active young fibroblasts or osteoblasts, and the elongated fibroblastoid cells as presumably fibroblasts or myofibroblasts.

While these characteristic stages together may not be seen in the same biopsy specimen, most histopathological descriptions agree on the presence of spindle cells embedded in interstitial collagen fibers and osteoclastic giant-cells. Hemosiderin deposition is frequently found [25,47]. Cytological examination after fine needle aspiration can identify stromal cells and giant-cells, but its usefulness for diagnosis is questionable [48,49] unless coupled with molecular analysis of *SH3BP2*.

## Psychosocial considerations and quality of life

The obvious concern of patients and their families is the facial disfigurement that is evident in cherubism. A second consideration is the possibility of genetic transmission to future children. Support groups (see below) and genetic counseling (see below) may help with management of these concerns. These supporting services function to augment the help available from family and friends. The shared experiences of other cherubism patients can be encouraging, e.g., the positive view on life of one severely affected patient published on the web site of the British Broadcasting Corporation (BBC): http://news.bbc.co.uk/2/hi/uk_news/magazine/3128203.stm.

## Diagnostic considerations and differential diagnosis

The diagnosis of cherubism is based on patient age, family history, clinical examination, radiographic findings, biochemical analyses and molecular analysis.

The classic clinical appearance of the cherubic face includes bilateral, symmetric, painless fullness of the cheeks and mandible in children at 2 to 7 years of age, when the diagnosis of cherubism is usually made. Retraction of the lower eyelids from bilateral mandibular and maxillary enlargement results in exposure of the sclera below the iris and an apparent upward gaze as described by Jones in 1933. However, this classic appearance is not infrequently absent and the patient may present with bilateral multicystic lesions which enlarge the mandible. These may be an incidental finding on radiographic examinations performed for other reasons such as trauma or during routine dental examinations.

Enlargement of the cervical lymph nodes that contribute to the patient’s full-faced appearance, a V-shaped palate with a high arch, early loss of primary teeth and displaced, impacted, supernumerary and missing teeth are common findings in patients with cherubism. Orbital involvement may appear late in affected individuals. This is manifest by osseous orbital expansion, globe displacement, proptosis, diplopia, optic neuropathy and loss of vision [18,20,50].

There have been reported cases of cherubism with massive enlargement of the jaws and backward displacement of the tongue resulting in airway obstruction and obstructive sleep apnea, speech, mastication and swallowing problems [21,22]. Some patients with severe cherubism report episodic pain [17,22,23,51,52]. Patients with aggressive, rapidly progressing cherubism should be evaluated by a craniofacial team consisting of a surgeon (oral and maxillofacial surgeon, plastic surgeon, otolaryngologist), geneticist/genetic counselor, ophthalmologist, dentist/orthodontist and child psychologist/social worker and nurse.

**Figure 2:**
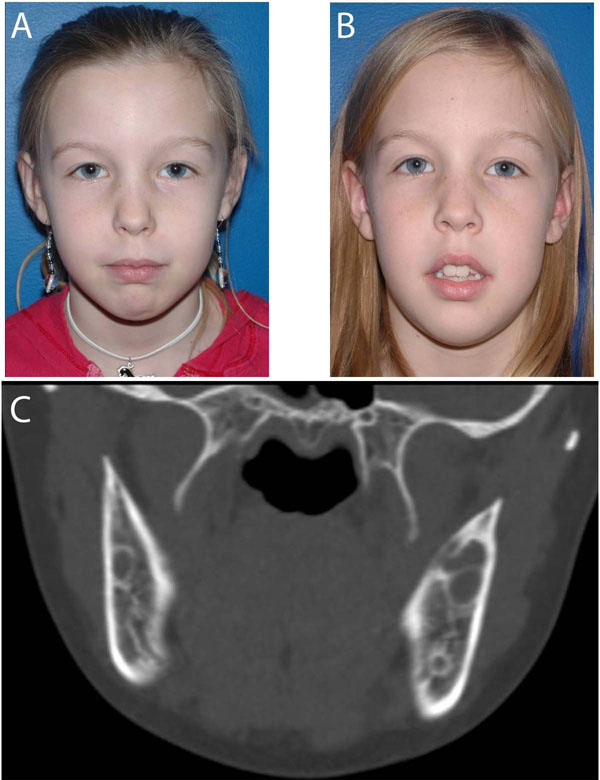
**A.** Photograph of a 10 year old girl with bilateral cheek and jaw swelling. Cherubism was confirmed with genetic testing that was positive for the SH3BP2 gene. The patient had genetic counseling and was followed longitudinally. **B.** Patient one year later with no progression of facial swelling. **C.** CT scan at initial presentation showed typical bilateral lytic lesions in the mandible that remained unchanged at follow ups.

At birth no signs of cherubism are present. Swelling of the jaws usually appears between 2 and 4 years of age. A rapid increase in size of the lesions and the affected jaws follows until the age of 7-8 years. After that, lesions remain unchanged or increase slowly until puberty (Figure 2). Around the age of puberty, the condition begins to regress and facial deformity starts to improve, although lesions can be still seen on radiographs. By 30 years of age, lesions are frequently not detectable. In a follow-up study of 18 patients with cherubism, von Wowern found progressive new bone formation in the lesions of patients over 20 years of age [27]. By 41 years of age, the bone structure in the affected areas was completely normal. Diagnosis in adults with a mild form of cherubism, not appreciated in childhood can be difficult as lytic bone cysts fill in with bone and may not be radiographically detectable. However, in rare instances actively expanding lesions in suspected cherubism may be diagnosed in adults [12].

Although not pathognomonic for cherubism, the radiographic findings of bilateral, multilocular, radiolucent areas within the mandible, usually located at the angles and rami, should raise the suspicion for the disease. The coronoid processes are commonly involved, whereas the condyles are rarely affected. Lesions in the mandible are usually symmetric, whereas those in the maxilla may be asymmetric [4]. Imaging typically shows expansile remodeling of the involved bones, thinning of the cortices, and multilocular radiolucencies with a coarse trabecular pattern.

Biopsy and histopathologic examination are not required in most cases to establish the diagnosis of cherubism. However, when performed, numerous osteoclast-like multinucleated giant cells in a moderately loose fibrous stroma are present. Thus cherubism is considered to be a fibro-osseous disorder. Ovoid to spindle shaped cells within the fine fibrillar collagenous stroma, numerous small vessels with large endothelial cells and perivascular capillary cuffing are also present. Eosinophilic cuffing appears to be specific to cherubism. However, these deposits are not present in many cases, and their absence does not exclude the diagnosis of cherubism [53]. The histological findings of cherubism are similar to those of aggressive or non-aggressive giant cell lesions, myxoma, aneurysmal bone cyst and hemangioma and other vascular lesions.

Gene testing is recommended to determine whether a mutation in the cherubism gene *SH3BP2* is present [54] and to confirm the clinical diagnosis of cherubism. For molecular analysis, genomic DNA from a blood sample or tissue from lesions is used for sequence analysis. Cherubism is an autosomal dominant disorder but most cases are due to *de novo* mutations. Therefore, the absence of a positive family history does not rule out the possibility of cherubism.

An important component in the management of cherubism is the differential diagnosis which includes brown tumor of hyperparathyroidism, giant cell lesions, Noonan/multiple giant cell lesion syndrome, fibrous dysplasia, aneurysmal bone cyst and the hyperparathyroidism-jaw tumor syndrome (HPT-JT). The limited and symmetrical distribution of the cherubism lesions can often facilitate distinction of cherubism from these other conditions, and of course mutation analysis of *SH3BP2* can confirm the diagnosis. If no mutation in *SH3BP2* is found cherubism cannot be excluded because of possible genetic heterogeneity.

Hyperparathyroidism may be differentiated by analysis of parathyroid hormone levels, calcium, phosphorous and alkaline phosphatase. However, hyperparathyroidism is rare in children except in the setting of chronic renal failure (secondary hyperparathyroidism). Fibrous dysplasia [55] and Noonan/multiple giant cell lesion syndrome [56-58] can be also be identified by genetic testing.

Noonan syndrome was first described in 1963 and is characterized by short stature, hypertelorism, prominent posteriorly angulated ears, congenital heart defect, low normal intelligence or developmental delay, cryptorchidism in males, and bleeding disorders [59]. In 1986, Chuong and colleagues published a series of 17 patients with giant cell lesions of the jaws studying the correlation of histologic appearance to biologic behavior [60]. Two of these patients had Noonan syndrome and bilateral giant cell lesions of the mandible and the maxilla. Dunlap et al. first reported on the Noonan syndrome and cherubism association and presented 4 children at age 4 to 8 years old with the combination of the two entities [61]. They considered the 2 patients with Noonan syndrome reported by Chuong et al. having cherubism as well. Later, in 1991, Cohen and Gorlin reviewed 15 cases with Noonan syndrome and giant cell lesions and proposed the name Noonan-like/multiple giant cell lesion syndrome and considered it to be separate from Noonan syndrome and cherubism [62]. Following that, 5 more cases of the Noonan-like/multiple giant cell lesion syndrome were published [63-65].

**Figure 3:**
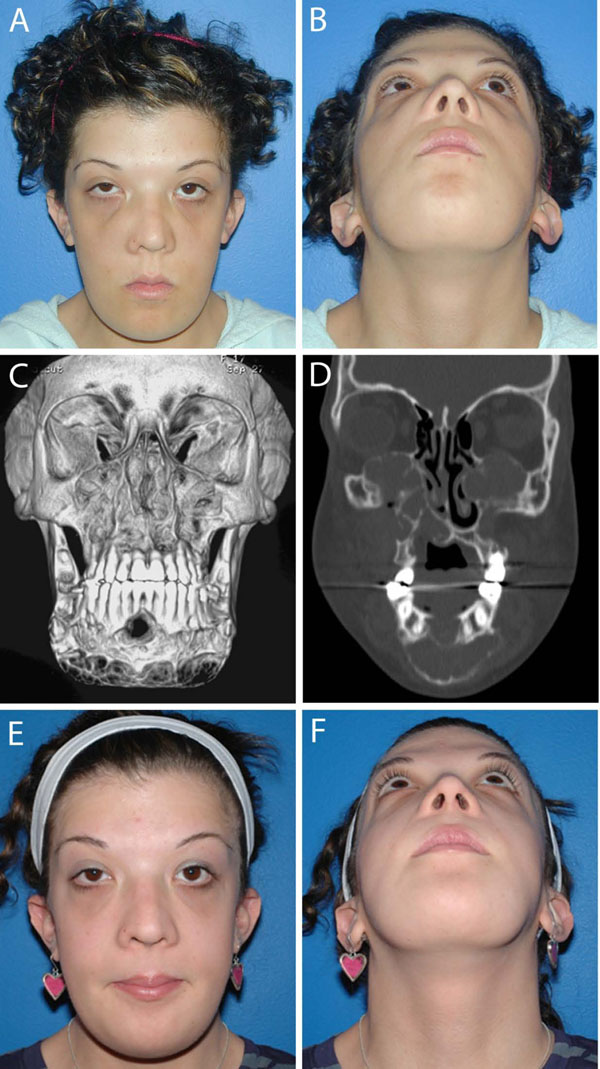
Noonan/multiple giant cell lesion syndrome. A 20-year old woman with Noonan/multiple giant cell lesion syndrome with bilateral involvement of the mandible and the maxilla. **A and B.** Frontal and submental photographs reveal prominent maxillary and mandibular contours, slight frontal bossing and increased intercanthal distance, downslanting palpebral features, epicanthal folds and posteriorly angulated ears. Lesions were painless to palpation. **C and D.** Coronal and 3D CT scan at initial presentation demonstrate involvement of the maxilla, including the maxillary sinuses and the nasal-maxillary region and the mandible from the antegonial notch to antegonial notch. The lesions are expansile and mixed radiolucent - radiopaque with cortical thinning and expansion into the orbits bilaterally. Patient was positive for a Noonan syndrome mutation in *SOS-1*. She underwent a contour resection of the maxillary and the mandibular giant cell lesions in two sessions. Postoperatively, she was treated with 4 courses of zoledronic acid, given at 6-week-intervals. **E and F.** Post-treatment frontal and submental photographs.

**Figure 4:**
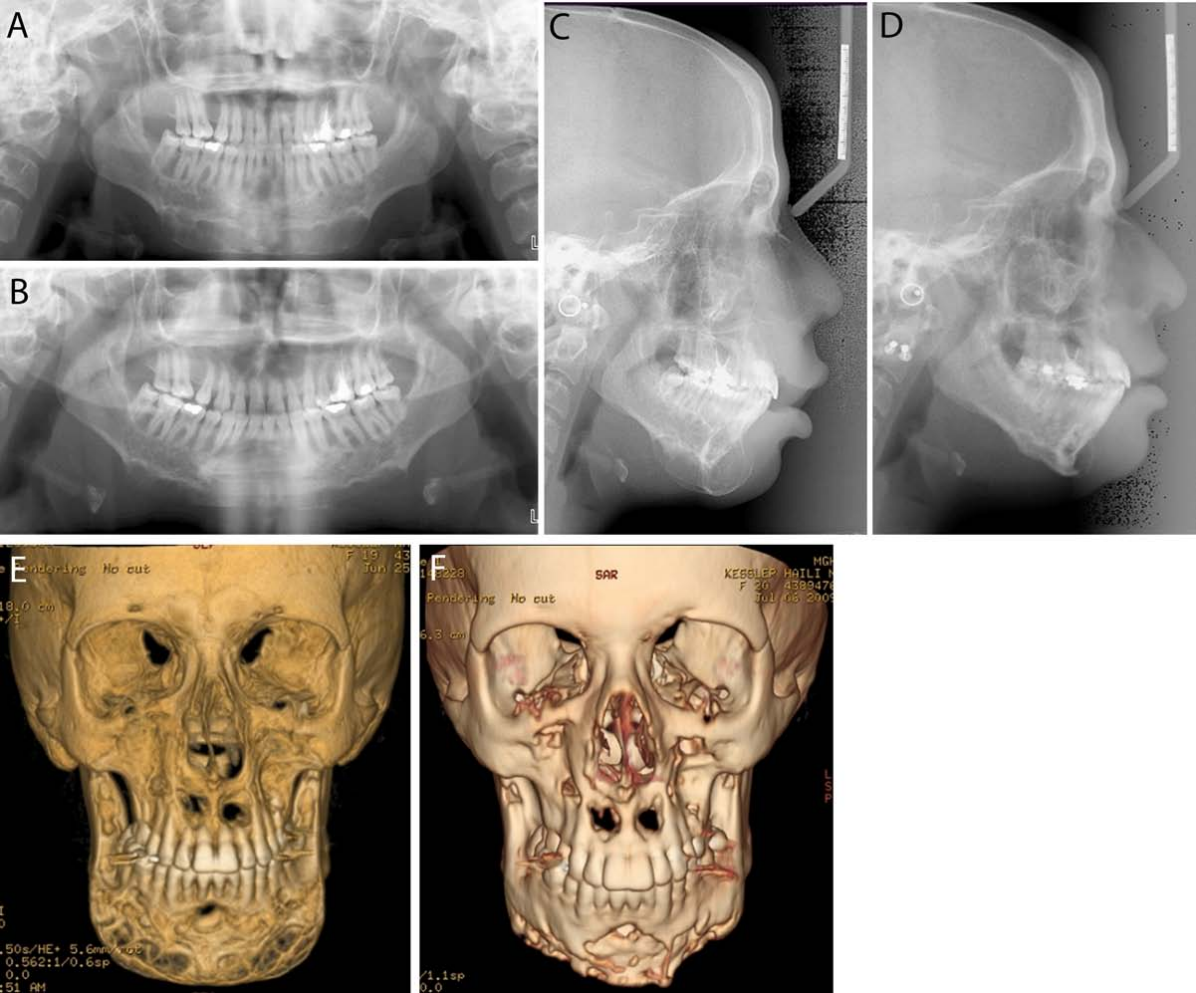
Imaging studies of the patient in Figure 3 pre-treatment (A, C, E) and post-treatment (B, D, F). **A and B.** Panoramic radiographs. **C and D.** Lateral cephalograms. **E and F.** 3D CT scans. Pre- and post-treatment images show improvement of the giant cell lesions and the contours of the mandible.

Later, mutations of the *PTPN11* gene [56-58,66] and the *SOS1* gene [67] were identified in patients with Noonan syndrome. These findings support the notion that the giant cell lesions in patients with Noonan syndrome are distinct from cherubism. Molecular analysis has led to the consideration of the Noonan syndrome with multiple giant cell lesions as a variant within the Noonan syndrome spectrum [57,67] and the term Noonan-like/multiple giant cell lesion syndrome should no longer be used. Rather Noonan/multiple giant cell lesion syndrome is more appropriate. There has also been a report of bilateral mandibular lesions in association with neurofibromatosis and a mutation in the *NF1* gene, which is associated with neurofibromatosis and with Noonan Syndrome [68]. Differentiation between cherubism and Noonan/multiple giant cell lesion syndrome is important as giant cell lesions may behave aggressively in the latter and can lead to considerable morbidity if not treated appropriately [69]. Giant cell lesions in Noonan patients can easily be mistaken for cherubism if the lesions appear symmetrically in maxilla and mandible (Figures 3 &4).

Cases of cherubism associated with other disorders such as fragile X syndrome, gingival fibromatosis with psychomotor retardation, neurofibromatosis type 1, and craniosynostosis have been published in the literature [15,70-72]. Finally, cherubism has been reported to be associated with Ramon syndrome [73] and Jaffe-Campanacci syndrome ([61]. Ramon syndrome is extremely rare with only 8 cases reported in the literature and presents with mental retardation, short stature, gingival fibromatosis and epilepsy [73,74]. Similarly rare is Jaffe-Campanacci syndrome [75], which includes non-ossifying fibromas that can be localized in long bones and/or jaw bones, mental retardation, *café au lait* spots, hypogonadism, ocular and cardiovascular anomalies (reviewed in [76]. However, to our knowledge only eccentric or unilateral mandibular or maxillary lesions have been described in the literature for Jaffe-Campanacci syndrome [61,77].

## Genetic origin, testing and counseling

The familial form of cherubism occurs typically in an autosomal dominant trait with mutations in the *SH3-domain binding protein 2* (*SH3BP2*) [54] on chromosome 4p16.3 [78,79]. However, approximately 50% of all cases of cherubism with mutations in *SH3BP2* identified at the University of Connecticut Health Center (UCHC) had no family history and were assumed to be *de novo* mutations. Germline mosaicism in parents is rare and while we cannot exclude this possibility, germline mosaicism is highly unlikely to account for these cases. In a few instances patients have been clinically diagnosed with cherubism but no mutation in *SH3BP2* has been found, suggesting the possibility of genetic heterogeneity. An autosomal recessive mode of inheritance has been suggested in some instances where signs of cherubism could not be found in carriers of the older generation [11]. It is important to note that the expressivity of cherubism is highly variable and mild forms of cherubism may be undiagnosed. Diagnosis of cherubism in older patients where a mild phenotype has been missed may not be possible as cherubic lesions in adults fill with normal mandibular bone and may no longer be detected by radiographs [27]. These considerations put reports of reduced penetrance in females into question [8,80].

Gene testing for known mutations in the *SH3BP2* gene [9] is offered by several commercial reference laboratories and testing on a research basis is available (see GeneTests: http://www.ncbi.nlm.nih.gov/sites/GeneTests/?db=GeneTests for updated information). Testing for a cherubism mutation may help to confirm the diagnosis.

Counseling by a medical geneticist or genetic counselor is recommended if family members are concerned that they may have cherubism. A gene test may resolve the concern if a mutation has been identified in the proband. Siblings of patients should be evaluated by physical examination, panoramic radiographs and genetic testing. Updated information about prenatal testing and preimplantation testing is available at GeneTests (http://www.ncbi.nlm.nih.gov/sites/GeneTests/?db=GeneTests).

## Management and treatment

Mild forms of cherubism without facial dysmorphology, dental and ocular involvement may not require treatment as cherubism is expected to regress spontaneously after puberty (Figure 2). Management in these cases consists of longitudinal observation. During the growth phase of the lesions, annual clinical and radiographic examination with a panoramic or other appropriate radiograph is suggested. Follow-up every 2 to 5 years is advisable after the disease becomes quiescent. Expansion of fibrous lesions in severe cases may regress well after adolescence [6,11,27,53,81,82] . In other instances actively expanding lesions in suspected cherubism may still be diagnosed in adults [12].

Surgical intervention is indicated when aesthetic or functional concerns arise including nasal obstruction, proptosis or facial deformity. Options for surgical management include partial resection, contour resection, curettage or a combination of these [83]. Surgical procedures should be performed after puberty when the lesions are quiescent. Severe aesthetic or functional problems may justify intervention prior to puberty. Orbital surgery may be required in rare cases when tumor tissue invades the floor of the orbits to a degree where it leads to the displacement of globes or loss of vision is suspected due to optic atrophy [19,50].

**Figure 5:**
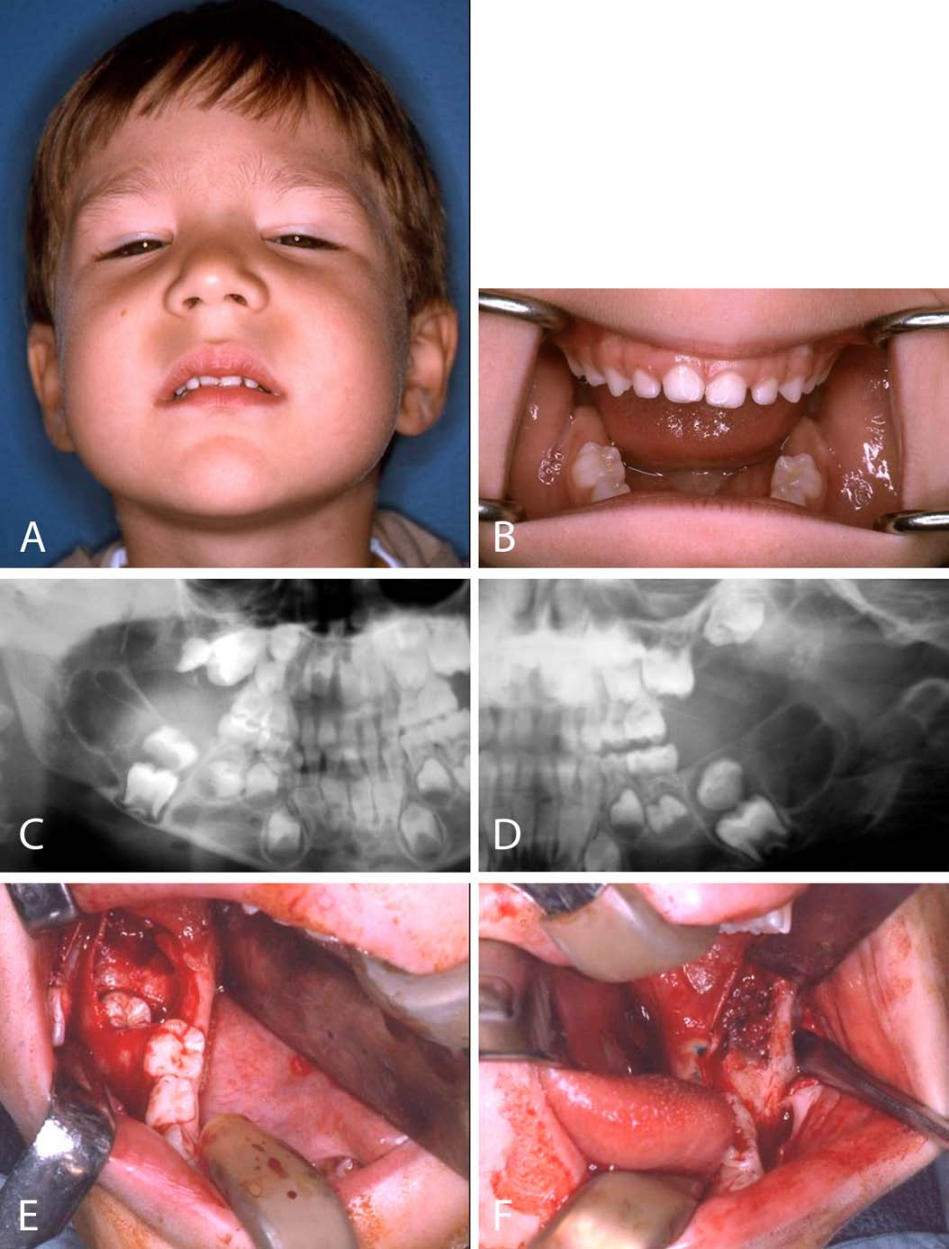
Cherubism. A 5 year old boy who after a mild trauma underwent a dental evaluation. **A.** Examination revealed marked fullness of the cheeks, expansion of the malar eminences and the mandibular angle regions. **B.** Intraoral examination was significant for expansion of the mandibular alveolus in the permanent molar regions. **C and D.** Panoramic radiograph revealed bilateral radiolucent lesions in the mandible and maxilla (arrows). The lesions were displacing the permanent second molar tooth buds superior to the first molar teeth. **E and F**. Intraoral biopsy and removal of the displaced second molar tooth buds leaving the first molar tooth buds in place to erupt. Pathologic diagnosis was fibro-osseous lesion consistent with cherubism.

Development of dentition should always be closely monitored. Roots may resorb or tooth displacement can occur in cases where lytic lesions develop around erupting secondary dentition or teeth may appear missing or to float in the lesion [11]. The problem of early loss of deciduous teeth, absence, delayed development or eruption of the permanent teeth is difficult to treat and no satisfactory solution is available. Space maintainers are used while waiting for the permanent teeth to erupt. Malocclusion is a major concern. Surgical exposure of impacted teeth is sometimes necessary (Figure 5) [81].

Because the expressivity of cherubism is variable, even within families, it is not surprising that reports of surgical outcome are variable as well. In a few studies, surgical contouring during the growth phase was associated with rapid regrowth of the tumor [84,85]. In addition, Shah et al. reported a case of leiomyosarcoma that arose in the mandible of a 10-year-old child with cherubism after two surgical recontouring procedures [39]. Favorable results after curettage and recontouring performed during the growth phase of cherubism have been reported as well. Dukart et al. reported one case of cherubism in which surgical intervention arrested active growth of remnant lesions while stimulating bone regeneration [86]. Von Wowern reported 18 patients with cherubism who underwent biopsy with or without autotransplantation of ectopically erupted teeth. Surgical treatment did not provoke progression of the lesions in any of these cases [27], which is consistent with other reports [40,41].

Raposo-Amaral and colleagues [7] reported on extensive resection in 8 children age 6 to 15 years old with severe cherubism. Surgical resection during the proliferating phase of the disorder was performed in 2 stages to prevent excessive blood loss. The maxilla and orbits were contour-resected first and the mandible 6 months later through intraoral and extraoral incisions. Patients were followed for 2 to 18 years and no recurrence was found in any of the patients. Patients and authors were satisfied with the outcome and the authors suggest that thorough removal of affected tissue appears to arrest the proliferation of any remaining tumor tissue.

Radiation therapy has been described in the literature for the management of cherubism. However, radiation therapy is contra-indicated in this benign condition because of the potential for long-term adverse consequences such as retardation of jaw growth, osteoradionecrosis and increased incidence of induced malignancy [53,84].

Calcitonin has been used for the treatment of central giant cell granuloma (CGCG) with successful outcome [87] and experimental use of calcitonin for the treatment of cherubism has been suggested [37]. In an 11-year-old boy with bimaxillary cherubism, lesions regressed and normal mandibular contour was restored with administration of salmon calcitonin nasal spray daily for 15 months [88].

However, Lannon and Earley treated a 7-year-old boy with significant mandibular enlargement and facial deformity due to presumed cherubism with calcitonin for 6 month without any visible effect [53]. The effect of calcitonin in giant cell granulomas can be seen after prolonged treatment (up to 18 months) and thus, calcitonin treatment would not be preferred for rapidly growing lesions [89,90]. Further studies to document the efficacy of calcitonin in the treatment of cherubism are required before it can be recommended as a conventional therapy.

Kaban et al. reported the first use of interferon for management of aggressive giant cell lesions in 1999 [91]. This group has also used a combination of contour resection and adjuvant interferon therapy for management of giant cell lesions in Noonan-multiple giant cell lesion syndrome. It is possible interferon could work in the proliferating phase of cherubism when vascular proliferation and multiple giant cells are present. Further investigation is warranted.

## Prognosis

Most cases of cherubism regress spontaneously after puberty. Carvalho Silva and colleagues describe that the cherubism lesions in 7 of 8 of their patients stabilized by age 12 years and regressed thereafter [92]. One more severely affected patient showed features of cherubism at age 20. Radiographic examination at follow-up visits revealed filling of the radiolucent lesions with bone as early as 2 years after stabilization and in most patients when they were in their twenties. In some cases the radiolucencies were replaced with sclerotic bone later in life. In more severe cases radiolucencies can remain. Corrective surgery may be performed after cherubism lesions become quiescent to achieve facial features that are acceptable to the patient. It is interesting to note that spontaneous fracture of jaw bones, even in severely affected patients, is not reported [7].

## Future perspectives (treatment modalities based on current research)

Ongoing research strongly suggests that abnormal inflammatory responses are an important component of the pathophysiology of cherubism. Research in a mouse model suggests that high levels of tumor necrosis factor-α (TNF- α) in the circulating blood system contribute to the progression of cherubism [9,28] A therapy to reduce TNF- α levels could be possible if this hypothesis holds true in humans as well. Such TNF- α blocker or antibody therapies have been approved for a number of other immune-mediated inflammatory diseases (reviewed in [93,94]). Although the mechanisms by which TNF- α antagonists work are not fully understood, it is conceivable, that reducing pro-inflammatory cytokine production and a reduction in osteoclast formation and resorption could have a positive effect on the rate of bone resorption and cherubism tissue expansion. However, experimental treatment with TNF- α blocking therapy in combination with bisphosphonates in a single patient showed no improvement [95]. The rate of progression during treatment and 6 month after treatment was considered the same.

More potential therapeutic targets will arise as molecular mechanisms for the action of mutant SH3BP2 become known. For example, the central role of the nuclear factor of activated T cells c1 (NFATc1) in regulating osteoclast gene expression makes this transcription factor another possible target for interfering with excessive bone resorption [96].

Ueki and coworkers hypothesize that – at least in mice – a hyper-active host response to oral pathogens or to physical damage may trigger the onset of cherubism [97]. Physical damage due to rapid bone remodeling for erupting secondary teeth could incite the bone resorptive lesions in cherubism. If this hypothesis holds true, new molecular targets for preventing cherubism in patients who have been diagnosed with a SH3BP2 gene mutation may be efficacious.

## Support groups

Several support and advocacy groups offer information and support for individuals affected with craniofacial disorders (Table 2). Updated information about cherubism may also be obtained from websites such as GeneTests.

**Table 2:** List of organizations providing information on cherubism

GeneTests at NCBI	http://www.ncbi.nlm.nih.gov/sites/GeneTests/?db=GeneTests (Pagon RA, Bird TC, Dolan CR, et al., editors. GeneReviews [Internet]. Seattle (WA): University of Washington, Seattle; 1993- )
National Library of Medicine Genetics Home Reference	http://ghr.nlm.nih.gov/condition=cherubism
Fibrous Dysplasia Foundation	http://www.fibrousdysplasia.org/
AboutFace International	http://www.aboutfaceinternational.org
Children's Craniofacial Association (CCA)	http://www.ccakids.com

## Conclusions

Although rare, cherubism has a significant impact on affected children and their families. This is especially true in those cases where aggressive growth leads to facial deformity and functional problems. In the majority of cases, cherubism is self-limiting and no surgical treatment is necessary apart from longitudinal clinical and radiographic observation, which should continue into adulthood. In cases of rapidly proliferating cherubism with significant functional consequences, resection may be indicated. Operative intervention does not change the disease progression but may improve function and appearance.

## Competing interests

The authors declare that they have no competing interests.

Written consent for publication was obtained from patients or their relatives.

## Authors’ contributions

This review is part of the Proceedings of the International Meeting on Fibrous Dysplasia/McCune-Albright Syndrome and Cherubism. MP, SL, LBK and EJR were involved in drafting the manuscript. All authors were involved in the critical review of the manuscript. All authors read and approved the final manuscript.
